# New Classification Method for Independent Data Sources Using Pawlak Conflict Model and Decision Trees

**DOI:** 10.3390/e24111604

**Published:** 2022-11-04

**Authors:** Małgorzata Przybyła-Kasperek, Katarzyna Kusztal

**Affiliations:** Institute of Computer Science, University of Silesia in Katowice, Bȩdzińska 39, 41-200 Sosnowiec, Poland

**Keywords:** Pawlak conflict analysis model, independent data sources, coalitions, decision trees, dispersed data

## Abstract

The research concerns data collected in independent sets—more specifically, in local decision tables. A possible approach to managing these data is to build local classifiers based on each table individually. In the literature, many approaches toward combining the final prediction results of independent classifiers can be found, but insufficient efforts have been made on the study of tables’ cooperation and coalitions’ formation. The importance of such an approach was expected on two levels. First, the impact on the quality of classification—the ability to build combined classifiers for coalitions of tables should allow for the learning of more generalized concepts. In turn, this should have an impact on the quality of classification of new objects. Second, combining tables into coalitions will result in reduced computational complexity—a reduced number of classifiers will be built. The paper proposes a new method for creating coalitions of local tables and generating an aggregated classifier for each coalition. Coalitions are generated by determining certain characteristics of attribute values occurring in local tables and applying the Pawlak conflict analysis model. In the study, the classification and regression trees with Gini index are built based on the aggregated table for one coalition. The system bears a hierarchical structure, as in the next stage the decisions generated by the classifiers for coalitions are aggregated using majority voting. The classification quality of the proposed system was compared with an approach that does not use local data cooperation and coalition creation. The structure of the system is parallel and decision trees are built independently for local tables. In the paper, it was shown that the proposed approach provides a significant improvement in classification quality and execution time. The Wilcoxon test confirmed that differences in accuracy rate of the results obtained for the proposed method and results obtained without coalitions are significant, with a *p* level = 0.005. The average accuracy rate values obtained for the proposed approach and the approach without coalitions are, respectively: 0.847 and 0.812; so the difference is quite large. Moreover, the algorithm implementing the proposed approach performed up to 21-times faster than the algorithm implementing the approach without using coalitions.

## 1. Introduction

In today’s world, data are often collected in a decentralized and dispersed manner. There are many examples that illustrate this process: hospitals that separately collect data on the same issue/disease; banks that store data on their clients; applications on mobile devices that collect various data. These data are collected independently and in separate data storage.

It is crucial to use these data sets simultaneously to construct a classification of new objects. Of course, a very significant consideration is to guarantee high efficiency in the classification process based on dispersed data.

The issues of dispersed data are mainly considered in distributed learning approaches [1,2]. The distributed models process all or part of the data at different nodes [3,4]. A solution in which all the data are simultaneously aggregated and stored in a single set is both inefficient and often impossible to apply [5]. Therefore, most research papers have proposed a collaborative solution without data aggregation. In federated learning [6,7], nodes perform multiple rounds with local data and send the local model to the central server for aggregation into new global models. The main idea here is to guarantee data protection and privacy. Moreover, models are much shorter than raw data, so the exchange of data is faster and less complex. In the distributed learning approach, methods can be found in which local models are built independently, and the final decision is simply generated by applying fusion methods. Various models have been proposed, both parallel [8] and hierarchical [9,10]. The concept of agent collaboration is also key here [11]; however, we do not build aggregated tables as a result of this collaboration. In the literature, examples of classifier ensembles in which feature subsets are considered can be found [12,13,14]. There are also ensembles of classifiers built based on subsets of objects [15,16]. In the paper [17], an approach that considers missing values in the context of ensembles is considered. A crucial matter that affects the quality of classification is diversity among the base classifiers [18,19]. The method for generating the final decision also has a significant impact on the efficiency of ensembles [20,21]. Approaches recognizing relations between local data are considered in the literature. In the paper [22], a hierarchical federated learning approach was proposed. On the other hand, the paper [23] proposed a hierarchical approach in classifier ensembles. Mainly in the literature, distributed learning is considered in terms of the following issues [2,24]: data division—horizontal or vertical fragmentation; type of base classifiers—can be homogeneous or heterogeneous; type and cost of communication—data or models may be shared; privacy and data security—whether raw data exchange is allowed; fusion methods—if local models are built (global model is not created) then fusion of predictions is necessary to generate global decisions; data consistency—it can be assumed that objects are shared between local tables and are consistent, or data can be independently created and inconsistent. However, proposed approaches do not analyze the contents of local tables and the relationships between them. In addition, the aggregation of local tables is seldom considered in the literature.

Therefore, in this paper we fill this gap and propose a solution that performs a complex analysis of tables’ content. The proposed approach aims to identify conflicts of local tables. The term conflict used here refers to significant differences in the values of conditional attributes occurring in local tables. We analyze relations and create coalitions of local tables containing similar data. Based on the aggregated tables, a model is built. It is expected that in this way we achieve better classification accuracy because models created via this approach have a better ability to generalize concepts compared to approaches that use a single model created based on a single table.

In the literature, conflict analysis is widely considered and various models are proposed. Group decision-making represents an approach that solves the situation in which each individual has their own private perspective [24]. In [25], a model is proposed for distributed group-decision support system that is suitable for use over the Internet. The theory of negotiation and coalition formation presents an important issue regarding social interaction and is also studied in computer science in the context of distributed systems [26,27]. Pawlak’s conflict analysis model [28,29] is yet another approach to conflict recognition that provides excellent solutions in a variety of applications [30,31]. Pawlak conflict analysis model was also considered in the context of dispersed data in the papers [32,33,34]. This application shows that the Pawlak model provides excellent results for dispersed data when tables are aggregated within coalitions. However, the approach discussed in their study is completely different from the one proposed in this paper. Here, the compatibility of tables is examined in terms of the information stored in them—the values on the attributes. In contrast, the papers [32,33,34] consider compatibility in terms of predictions generated by the base models created based on the tables. Another difference is that in this paper we assume that in local tables the same attributes are present, while in the papers [32,33,34] there was no such assumption. Furthermore, in this paper, the system is static, whereas previously it was dynamic. However, the success of the previous model provides the inspiration for proposing a new approach in this paper. The main differences between these approaches are listed in Table 1.

This paper proposes the use of the Pawlak conflict analysis method to generate coalitions of decision tables, in which there are similar values on a set of conditional attributes. The goal is to achieve a better quality of classification by ensuring that similar units work together. Formally, this approach requires that data are collected in a set of decision tables (that were collected independently) in which the names of the conditional attributes are identical (but the values on the objects may differ). Thus, coalitions of tables containing similar values will be created. The tables in one coalition are then aggregated and a common model is determined based on the aggregated table. This approach seems natural, since in everyday life we also notice that similar entities join forces to form better decisions or to guarantee better management. This paper describes the process of using characteristics of attribute values stored in decision tables in the Pawlak conflict analysis model. The paper proposes a static and hierarchical classification model. The model is static because coalitions—the model’s structure—are determined only once. Hierarchy of the model results from the fact that tables in coalitions are aggregated and then models are built based on them and these models perform classification. In this paper, decision trees are used as base models. Specifically, classification and regression trees with Gini index (CART) [35] are applied. The final classification of new objects is determined using majority voting based on the predictions generated by the decision trees.

The paper also considers a parallel approach in which conflict analysis is not considered. In this approach, the CART trees are also employed as base models, but the cooperation of tables is not implemented, and the final decisions are made by majority voting of decision trees generated independently based on tables.

The main objective in this study is to analyze how building coalitions of tables using the Pawlak conflict analysis model affects the quality of classification and the running time of the model. The two research hypotheses are verified in the paper. The first is that applying the proposed model with Pawlak analysis and coalitions provides better classification quality than an approach in which coalitions are not used (in both models the same base classifiers are used—the CART trees). The second research hypothesis is that the algorithm implementing the proposed model has a lower time complexity than the algorithm implementing the approach in which decision trees are built based on each local table separately.

Herein, it is shown that combining local tables into aggregated tables significantly improves classification quality. In addition, it reduces the number of generated trees and thus reduces the time complexity of the method.

The main contributions of the paper are:

- proposing a new classification model using cooperation and coalitions of local tables (tables contain the same attributes),
- proposing a new method for creating coalitions of tables using the Pawlak conflict analysis model,
- developing a hierarchical system with CART trees for classification based on dispersed data.

The structure of the paper is organized as follows. Section 2 presents the proposed model. The method of defining the coalitions and steps in building the model are described there. Section 3 is dedicated to presenting the experimental results. The data, the measures used and the methodology of the experiments are described in this section, and the results obtained are also provided in tables. Section 4 contains the discussion and comparisons of the obtained results. Section 5 gives conclusions and future research plans.

## 2. Materials and Methods

**Figure 1:**
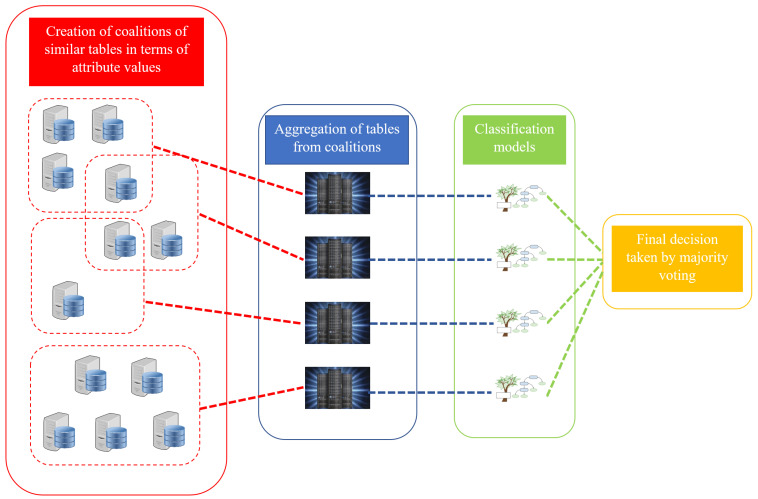
The overall workflow of the proposed model.

This section describes a new proposed hierarchical system for classification based on dispersed data. In this research, we assume that the sets of attributes appearing in local tables are equal. Stages of system construction are described in the following subsections. The first step involves creating the system’s structure—generating coalitions of local tables. This stage is implemented only once. Our goal here is the cooperation of tables that store similar conditional attribute values. This concept detailing the cooperation of units that share similar views with each other—have compatible values in this case—represents a natural behavior that we can observe in everyday life and nature. For this purpose, characteristics of conditional attributes’ values are calculated. In the next step, coalitions are created based on these characteristics using the Pawlak conflict analysis model. The final step is the aggregation of tables from one coalition. Based on such aggregated coalition’s data, a classifier is built. In this study, we use a decision tree model. The final classification model is a set of such decision trees generated for coalitions. The classification of an object is conducted by the majority voting of these trees. Figure 1 illustrates the workflow of the proposed model.

### 2.1. Basic Concepts and Method of Defining Characteristics of Conditional Attributes

We assume that a set of decision tables is given. The tables were collected independently by separate units, but it is required that the same attributes are stored in all tables. We do not impose any restrictions on the objects contained within the tables. We assume that we do not know which objects are shared between local tables.

Formally, we assume that a set of decision tables $D_{i}=\left(U_{i},A,d\right),i\in \left\{1,\ldots ,n\right\}$ from one discipline is available, where $U_{i}$ is the universe, a set of objects; *A* is a set of conditional attributes; *d* is a decision attribute. As can be seen the sets of objects are different between local tables. The names of attributes that occur in local tables, both conditional and decision, are the same. Therefore, the conditional attributes *A* and decision attribute *d* in all local tables are denoted in the same way. Clearly, from a formal point of view, the attribute a∈A in the decision table $D_{i}$ is a function $a:U_{i}\to V^{a}$, where $V^{a}$ is the set of values of the attribute *a*. Thus, the domains of the functions between local tables are different. However, for the sake of simplicity, the same designations for attributes were adopted in all local tables, and the domain of the function will be directly derived from the attribute’s membership in the decision table. Aggregation for these tables is a difficult process and can generate inconsistencies. Another aspect that should be taken into account is data protection and privacy. In addition, the process of aggregating all local tables is highly complex. Thus, in the literature, rather, methods are proposed for partial aggregation of tables or even building separate models based on each local tables, and then aggregating these models or the predictions generated by the models [7,21,36].

In this paper, a new approach is proposed in which we aggregate tables that contain similar values on conditional attributes. For this purpose, for each local table and for each attribute, some characteristics of the attribute’s values occurring in the table are generated. Suppose that in each local table we have *m* attributes card{A}=m (card denotes the number of elements in the set). Let us assume that we have $m_{1}$ quantitative attributes and $m_{2}$ qualitative attributes, so $m_{1}+m_{2}=m$.

For each quantitative attribute $a_{quan}\in A$, we determine the average of all attribute’s values present in local table $D_{i}$, for each i∈{1,…,n}. Let us denote this value as ${\bar{Val}}_{a_{quan}}^{i}$. We also calculate the global average and the global standard deviation. Let us denote them as ${\bar{Val}}_{a_{quan}}$ and $SD_{a_{quan}}$. These values are determined based on the averages calculated for the local decision tables according to the following formulas:

(1) $${\bar{Val}}_{a_{quan}}=\frac{1}{n}\sum_{i=1}^{n}{\bar{Val}}_{a_{quan}}^{i}$$

(2) $$SD_{a_{quan}}=\sqrt{\frac{1}{n}\sum_{i=1}^{n}{\left({\bar{Val}}_{a_{quan}}-{\bar{Val}}_{a_{quan}}^{i}\right)}^{2}}$$

These characteristics for quantitative attributes will be used in the coalitions generation process.

For each qualitative attribute $a_{qual}\in A$, we determine a vector over the values of that attribute. Suppose attribute $a_{qual}$ has *c* values $val_{1},\ldots ,val_{c}$. The vector $Val_{a_{qual}}^{i}=\left(n_{1}^{i},\ldots ,n_{c}^{i}\right)$ represents the number of occurrences of each of these values in the decision table $D_{i}$. More precisely, the coordinate $n_{j}$ represents the number of objects in table $D_{i}$ that have value $val_{j}$ on attribute $a_{qual}$. This vector is normalized. This is done to ensure that in further analysis the percentage of occurrences of a given value in the table matters rather than the number of objects in the table.

The Pawlak conflict analysis model is employed to determine coalitions of local tables that store similar attribute values. The next section presents the method to create an information system with a description of the conflict situation and how coalitions are generated with the use of the Pawlak model.

### 2.2. Pawlak Conflict Analysis Model and Creation of Coalitions

The Pawlak conflict analysis model is a very simple yet effective approach for recognizing coalitions of units involved in a conflicting situation [28,29]. In this model, an information system is defined in which the views of agents—units involved in a conflict situation—on the issues that are the matter of the conflict are stored. In the considered approach, the agents are local tables while the issues are conditional attributes stored in these tables. Formally, an information system is defined S=(U,A), where *U* is a set of local decision tables $U=\{D_{1},\ldots ,D_{n}\}$ and *A* is a set of conditional attributes (qualitative and quantitative) occurring in local tables, which was defined in the previous section. In the Pawlak model, opinions of agents on issues are expressed by using three values. Value 1 means an agent is in favor of an issue, value 0 means an agent is neutral to an issue, while value −1 means an agent is against an issue. The original interpretation differs from that used herein. In this paper, the values refer rather to the differences in values of a given attribute appearing in the local decision table. Depending on the type of attribute (qualitative or quantitative), a different method of determining these values is used.

For the quantitative attribute $a_{quan}\in A$ a function $a_{quan}:U\to \left\{-1,0,1\right\}$ is defined

(3) $$a_{quan}\left(D_{i}\right)=\left\{\begin{array}{cc} 1 & \mathrm{if}\;\;\;{\bar{Val}}_{a_{quan}}+SD_{a_{quan}}<{\bar{Val}}_{a_{quan}}^{i}\\ 0 & \mathrm{if}\;\;\;{\bar{Val}}_{a_{quan}}-SD_{a_{quan}}\leq {\bar{Val}}_{a_{quan}}^{i}\leq {\bar{Val}}_{a_{quan}}+SD_{a_{quan}}\\ -1 & \mathrm{if}\;\;\;{\bar{Val}}_{a_{quan}}^{i}<{\bar{Val}}_{a_{quan}}-SD_{a_{quan}} \end{array}\right.$$

The motivation for proposing this function originates from the method of estimating typical values of normal distribution. It is known that about 68% of the typical values from the normal distribution fall within the range: average ± standard deviation. Thus, we assign the value 0 on attribute $a_{quan}$ to decision tables $D_{i}$ when the average of the attribute’s values occurring in the table falls in the $SD_{a_{quan}}$-neighborhood of the global average ${\bar{Val}}_{a_{quan}}$.

This means that the values of the attribute occurring in the decision table are typical.

In contrast, the value 1 means that the average of the conditional attribute values in the decision table is above the global average more than $SD_{a_{quan}}$ value; it deviates more than the value of the standard deviation. Similarly, the value −1 indicates an atypical—lower—average value of the conditional attribute in the decision table compared to the global average value.

As mentioned above, the vectors that determine the distribution of values occurring in the decision tables are generated for qualitative attributes. For an attribute $a_{qual}\in A$ we have the vectors $Val_{a_{qual}}^{i}=\left(n_{1}^{i},\ldots ,n_{c}^{i}\right),i\in \left\{1,\ldots ,n\right\}$. In order to define three groups of decision tables with similar distribution of the attribute’s $a_{qual}$ values, we group these vectors with the k–means clustering algorithm, fixed number of groups k=3 and the Euclidean distance. We then place in descending order the centroids obtained for groups. Ordering with respect to the value of the first centroid coordinate was applied. Let us denote the groups of decision tables obtained from the k–means algorithm and indexed in relation to the centroids’ order as $G_{1},G_{2},G_{3}$. For the qualitative attribute $a_{qual}\in A$ a function $a_{qual}:U\to \left\{-1,0,1\right\}$ is defined

(4) $$a_{qual}\left(D_{i}\right)=\left\{\begin{array}{cc} 1 & \mathrm{if}\;\;D_{i}\in G_{1}\\ 0 & \mathrm{if}\;\;D_{i}\in G_{2}\\ -1 & \mathrm{if}\;\;D_{i}\in G_{3} \end{array}\right.$$

The function above assigns values on a qualitative attribute to local tables that reflect the consistency of the characteristics of this attribute appearing in the table. Thus, decision tables that contain similar distribution of values of the qualitative attribute will have the same value assigned in the information system *S*.

In this way, the information system *S* is defined that stores information about the compatibility of values of conditional attributes occurring in local tables. Based on this system, we calculate the general similarity of values of all attributes for each pair of tables. For this purpose, a conflict function is used that was proposed by Pawlak in their conflict analysis model [28]. The conflict function ρ:U×U→[0,1] is defined as follows

(5) $$\rho \left(D_{i},D_{j}\right)=\frac{card\{a\in A:a\left(D_{i}\right)\neq a\left(D_{j}\right)\}}{card\{A\}}.$$

A pair of decision tables $D_{i},D_{j}\in U$ is said to be [28]:

- allied, if $\rho (D_{i},D_{j})<0.5$,
- in conflict, if $\rho (D_{i},D_{j})>0.5$,
- neutral, if $\rho (D_{i},D_{j})=0.5$.

Set X⊆U is a coalition if for every $D_{i},D_{j}\in X$ decision tables are allied $\rho (D_{i},D_{j})<0.5$.

By applying the Pawlak conflict analysis model, we obtain coalitions of local tables that share similar values of conditional attributes. It should be noted that coalitions do not have to be disjointed—one local table can be included in several coalitions. In fact, this is a quite common case, as will be shown in the experimental section.

The pseudo-code of the algorithm that generates the coalitions of local tables is given in Algorithm 1.
**Algorithm 1:** Pseudo-code of algorithm generating coalitions of local tables**Input:** A set of local decision tables $D_{i}=\left(U_{i},A,d\right),i\in \left\{1,\ldots ,n\right\}$. **Output:** A set of coalitions of local tables $X_{1},\ldots ,X_{k}$. *Construction of an information system*
S=(U,A)*, where*
$U=\{D_{1},\ldots ,D_{n}\}$
*and*
*A*
*is a set of conditional attributes* for each a∈A:  if *a* is a quantitative attribute then    Use Equation  (3) to define the function *a*  else    Use Equation (4) to define the function *a* *Conflict function values* for each pair $D_{i},D_{j}\in U$:  Use Equation (5) to calculate the value $\rho (D_{i},D_{j})$ *Creation of coalitions*
$X_{1}=U,i=1,j=1$ while i≤j:  Repeat until there is a pair of tables $D_{l},D_{k}\in X_{i}$ so that $\rho (D_{l},D_{k})\geq 0.5$:    j=j+1    $X_{j}=X_{i}\backslash \left\{D_{l}\right\}$, $X_{i}=X_{i}\backslash \left\{D_{k}\right\}$  i=i+1 Return only the largest sets, due to the inclusion relation, from the sets $X_{i},i=1,\ldots ,j$

The computational complexity of the algorithm is exponential due to the number of local tables. The greatest complexity is noted when there exists no pair of local tables similar enough to satisfy the conditions of being allied. Subsequently, all subsets of the set of local tables will eventually be checked. However, in most applications, the number of local tables is not so large. In the experimental section, the application of the proposed model is checked for dispersed data containing up to eleven local tables. The obtained times in the worst cases are expressed in minutes.

### 2.3. Aggregation of Tables from Coalitions and Final Classification

An aggregated decision table is defined for each coalition of local tables generated in the previous step. Suppose we have coalitions of tables $X_{1},\ldots ,X_{k}$. The aggregated decision table for the coalition $X_{j}$ is denoted as $D_{j}^{aggr}=\left(U_{j}^{aggr},A,d\right)$, where $U_{j}^{aggr}={⋃}_{D_{i}\in X_{j}}U_{i}$ and the names of attributes in the aggregated table are the same as those in local tables. The attribute *a* from the aggregated table is a function defined on $U_{j}^{aggr}$ that takes values in $V^{a}$. The attribute *a* from the aggregated table has the same value, on object $x\in U_{i}$, as the corresponding attribute *a* from the local table $D_{i}$ on that object. Thus, an aggregated table is defined by summing objects from local tables in the coalition without recognizing whether there are common objects in the local tables (based on the assumptions, we do not possess this possibility). In the aggregated table, the values assigned to objects on the attributes are taken from local tables.

Based on aggregated tables, models are generated. In this paper, the classification and regression tree algorithm is used with Gini index [35]. It should be noted that prepruning and postpruning were not used for this tree. An implementation available in Python language was used for this purpose [37]. Specifically, *DecisionTreeClassifier(criterion = “gini”)* function was used. The tree is built independently for each aggregated table, thus we obtain *k* models $M_{1},\ldots ,M_{k}$.

The classification of a new object *x* is realized by each model separately. The final decision—the global decision, which we denote as $\hat{d}\left(x\right)$—is made by majority voting. This means that there may be a tie, which we do not resolve in any way. Thus, $\hat{d}\left(x\right)$ is the set of decisions that were most frequently indicated by models $M_{1},\ldots ,M_{k}$. In the experimental part, the relevant measures for evaluating the quality of classification, which takes into account the possibility of draws, were used.

In the section below, an illustrative example of the proposed approach is provided for clarification.

### 2.4. Baseline Model without the Use of Coalitions

The results obtained using the proposed method are compared with the results generated by an approach without any conflict analysis. In the baseline approach, a model is built based on each local table. In order to perform a fair comparison of the impact of the proposed novelty on the results obtained, the same classification model was used—for each local table the CART tree is used. Classification of a new object is realized by applying the majority voting method to the classification results obtained using these decision trees. Ties can occur, but as stated before, we do not resolve them in any way. The adequate measures were used in the experimental part.

### 2.5. Example of Use of the Proposed Approach

Let us consider an example that uses the proposed approach. Suppose we have a set of four local tables $D_{i}=\left(U_{i},A,d\right),i\in \left\{1,\ldots ,4\right\}$. Each of them contains a set of five conditional attributes $A=\{a_{1},\ldots ,a_{5}\}$ and a decision attribute *d*. We assume that $V^{a_{i}}=\left\{0,1,2\right\},i\in \left\{1,\ldots ,5\right\}$, and $V^{d}=\left\{d_{1},d_{2}\right\}$ for each of the tables. For the purposes of this example, the conditional attributes in the tables are quantitative. The local tables defined above are given in Table 2.

Based on the attribute values in the local tables (Table 2), the information system is generated as described in Section 2.2. In the first step, the average of all attribute’s values occurring in the local table for each attribute and each table is calculated. These values are denoted as ${\bar{Val}}_{a_{j}}^{i}$, i∈{1,…,4},j∈{1,…,5} and are given in Table 3. Furthermore, the global average and the global standard deviation for each attribute are calculated, the values are also shown in Table 3.

Thus, according to Equation (3), the values in the information system for attribute $a_{1}$ are assigned as follows

(6) $$a_{1}\left(D_{i}\right)=\left\{\begin{array}{cc} 1 & \mathrm{if}\;\;\;1.337<{\bar{Val}}_{a_{1}}^{i}\\ 0 & \mathrm{if}\;\;\;1.163\leq {\bar{Val}}_{a_{1}}^{i}\leq 1.337\\ -1 & \mathrm{if}\;\;\;{\bar{Val}}_{a_{1}}^{i}<1.163 \end{array}\right.$$

which means that $a_{1}\left(D_{1}\right)=0,a_{1}\left(D_{2}\right)=1,a_{1}\left(D_{3}\right)=0,a_{1}\left(D_{4}\right)=0,a_{1}\left(D_{5}\right)=0.$ For other attributes, the values in the information system are determined similarly. The obtained information system is shown in Table 4.

In the next step, the values of conflict function for the local tables are determined according to Equation (5). For example, for the pair $\left(D_{1},D_{2}\right)$ of local tables, the value is calculated as follows

(7) $$\rho \left(D_{1},D_{2}\right)=\frac{card\{a\in A:a\left(D_{1}\right)\neq a\left(D_{2}\right)\}}{card\{A\}}=\frac{4}{5}.$$

The values of the conflict function for the above information system are presented in Table 5.

**Figure 2:**
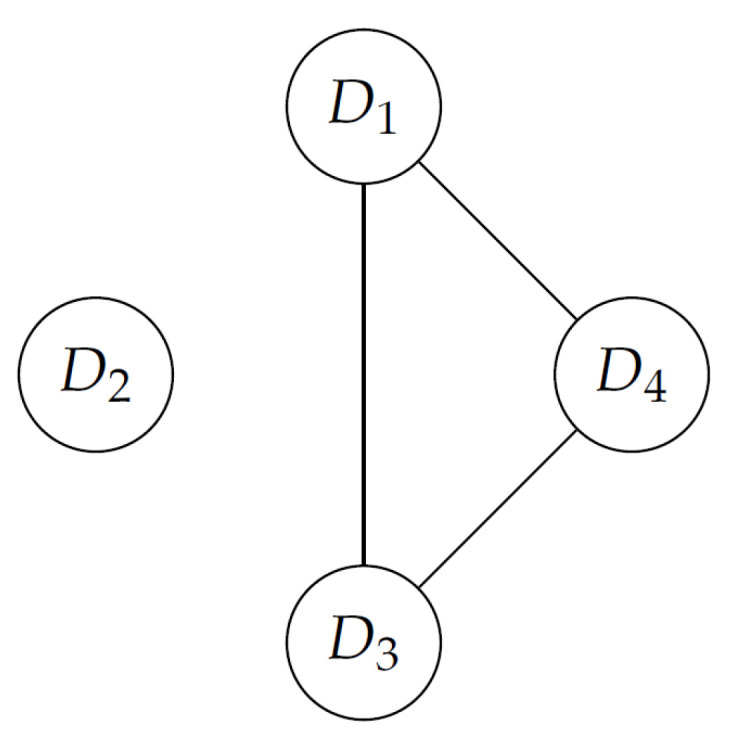
A graphical representation of the conflict situation example.

Figure 2 shows a graphical representation of the conflict situation. When agents (local tables) are allied $\left(\rho \left(D_{i},D_{j}\right)<0.5\right)$, the circles representing the agents are linked. In order to find coalitions, all cliques should be identified in the graph. In this example, there are two coalitions: $\left\{D_{1},D_{3},D_{4}\right\}$ and $\left\{D_{2}\right\}$.

An aggregated decision table is generated for each coalition. The aggregated tables are presented in Table 6.

**Figure 3:**
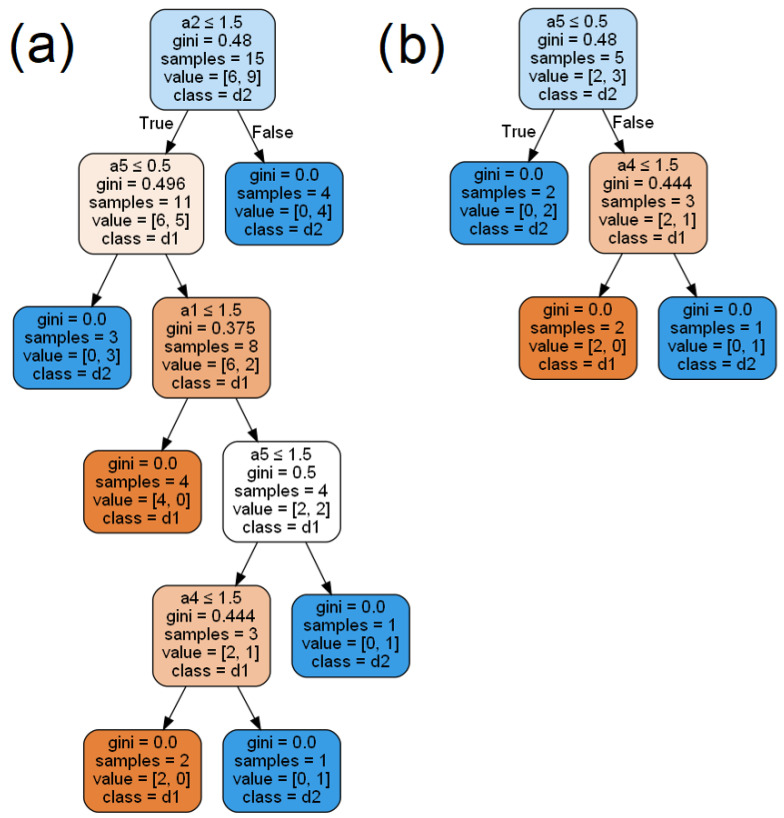
Decision trees created for aggregated decision tables. (**a**) The aggregated table ${D_{1}}^{aggr}$ (**b**) The aggregated table ${D_{2}}^{aggr}$.

Now, a decision tree is built for each aggregated table. This is done using the function implemented in the Scikit-learn library *tree.DecisionTreeClassifier(criterion = ”gini”)*. The built decision trees are presented in Figure 3. Test objects are classified based on these models using the simple voting method.

**Figure 4:**
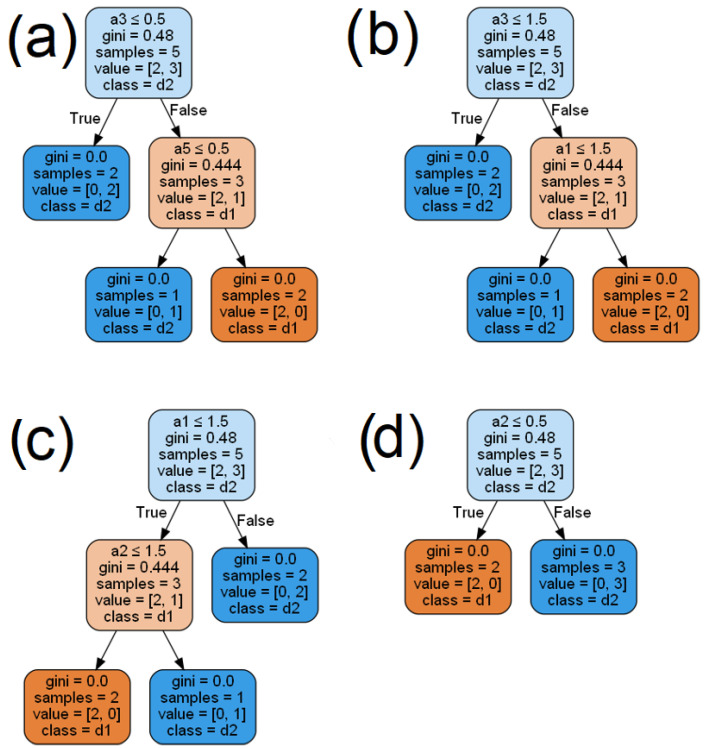
Decision trees created for local decision tables, (**a**) for the local table $D_{1}$, (**b**) for the local table $D_{2}$, (**c**) for the local table $D_{3}$, (**d**) for the local table $D_{4}$.

Since local table $D_{2}$ is left in a coalition containing only one element, the second aggregated table is the same as the local table $D_{2}$, therefore, the trees generated based on them are also the same. So we should mainly focus on the tree generated based on the first aggregated table and the three trees generated from local tables $D_{1}$, $D_{3}$ and $D_{4}$. As we can see, they are quite different. For example, in the tree generated based on the aggregated table there is a condition $a_{2}\leq 1.5$ the root, which does not correspond to the conditions occurring in the trees in Figure 4a,c,d. In addition, in the aggregated tree, there is the attribute $a_{5}$ in two internal nodes and the attribute $a_{4}$ in one internal node. These attributes are not included at all in the trees generated from local tables $D_{1}$, $D_{3}$ and $D_{4}$.

Since tables are combined into coalitions in terms of similarity of conditional attributes’ values, trees generated based on aggregated tables should not be very altered compared to trees generated from local tables. In general, trees generated from a larger number of training objects are expected to be more accurate and have better classification quality.

For comparison, let us also consider the baseline model, in which coalitions are not generated. In this case, the decision trees are generated directly based on local tables. Thus, we obtain four decision trees generated from the tables given in Table 2, which are presented in Figure 4.

## 3. Results

The experiments were carried out using the data available from the UC Irvine Machine Learning Repository [38]. A total of three data sets were selected for the analysis—the Vehicle Silhouettes, the Landsat Satellite and the Soybean (Large) data sets. Regarding the Landsat Satellite and Soybean data sets, the training and test sets are located in the repository. The Vehicle data set was randomly split into two disjoint subsets, the training set (70% of objects) and the test set (30% of objects). Data characteristics are given in Table 7.

The training sets of the above data sets were dispersed. A total of 5 different dispersed versions with 3, 5, 7, 9 and 11 local tables were prepared to check for different degrees of dispersion for each data set. This was done using a stratified mode. Each local table contained the full set of attributes, and a subset of the set of objects.

The quality of classification was evaluated based on the test set. The following measures were used:

- the classification accuracy
  $$acc=\frac{1}{card\{U_{test}\}}\;\sum_{x\in U_{test}}I\left(d\left(x\right)\in \hat{d}\left(x\right)\right),$$
  where $I(d\left(x\right)\in \hat{d}\left(x\right))=1$, when $d\left(x_{i}\right)\in \hat{d}\left(x\right)$ and $I(d\left(x\right)\in \hat{d}\left(x\right))=0$, when $d\left(x\right)\notin \hat{d}\left(x\right)$; $\hat{d}\left(x\right)$ is a set of global decisions generated by the system for the test object *x* from the test set $U_{test}$
- the classification ambiguity accuracy
  $$acc_{ONE}=\frac{1}{card\{U_{test}\}}\sum_{x\in U_{test}}I\left(d\left(x\right)=\hat{d}\left(x\right)\right),$$
  where $I(d\left(x\right)=\hat{d}\left(x\right))=1$, when $\left\{d\left(x\right)\right\}=\hat{d}\left(x\right)$ and $I(d\left(x\right)=\hat{d}\left(x\right))=0$, when $\left\{d\left(x\right)\right\}\neq \hat{d}\left(x\right)$
- the average size of the global decision sets
  $$\bar{d}=\frac{1}{card\{U_{test}\}}\sum_{x\in U_{test}}card\left\{\hat{d}\left(x\right)\right\}.$$

The classification accuracy refers to the ratio of correctly classified objects from the test set to their total number in this set. When the correct decision class of an object is contained within the generated decision set, the object is considered to be correctly classified. The classification ambiguity accuracy also describes the ratio of correctly classified objects from the test set to their total number in this set. With the difference being that this time when only one correct decision class is generated, the object is considered to be correctly classified. The third measure allows us to assess the frequency and number of draws generated by the classification model.

The experiments were conducted according to the following scheme:

- Generating coalitions of local tables using the Pawlak conflict analysis model. Detailed information on the coalitions that were generated is shown in Table 8. In cases where no coalitions were generated for a set of local tables then the dispersed set was not considered for further analysis. The reason for this is that the data in the tables are so different that they should not be combined and the proposed model does not bring any changes compared to the baseline approach.
- Defining aggregated tables for coalitions and generating decision tree models based on them. The classifier is a set of decision trees generated based on the aggregated tables for coalitions. Evaluating the proposed model using a test set.
- Analysis of the baseline approach. Generating decision trees based on the local tables (without any conflict analysis or coalitions). The final decision is made by simple voting. Evaluating the baseline approach using a test set.

As mentioned above, Table 8 shows the coalitions generated during construction of the proposed model. As can be seen, in two cases no coalitions were generated—for the Satellite and Soybean data sets with three local tables. In most cases, coalitions were created and, as can be seen, they are not disjoint sets. This means that some local tables were involved in the creation of several aggregated tables. The reason for this is that a given local table is partially similar to different sets of local tables and provides additional knowledge to the construction of trees representing different concepts.

Table 9 presents the classification accuracy acc values, the classification ambiguity accuracy $acc_{ONE}$ values and the average number of generated decisions set $\bar{d}$ obtained for all dispersed data sets. The table shows the results obtained for both the proposed approach and the baseline approach. For each data set, the better result is indicated in bold. As can be seen, in the vast majority of cases better results are generated by the proposed model with creation of coalitions and recognition of similarity of data stored in local tables.

**Figure 5:**
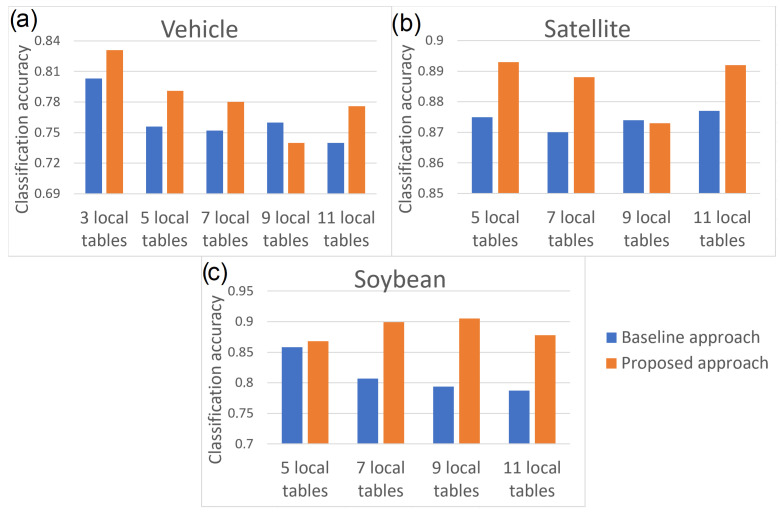
Comparison of classification accuracy (*acc*) of the baseline approach versus the proposed approach: (**a**) the Vehicle data set (**b**) the Landsat Satellite data set (**c**) the Soybean data set.

To better visualize the differences in the results generated by the models, Figure 5 was prepared with the classification accuracy marked for each data set. As can be seen, the most significant improvement in classification quality using the proposed approach was observed for the Soybean data set. Here, the improvement is around 0.1. For the Vehicle Silhouettes data set, the improvement in most cases is around 0.03 (even greater in certain scenarios). Furthermore, for the Landsat Satellite data set, the improvement in results was also noticed, but smaller at around 0.015. However, for all data sets, there is a noticeable and seemingly significant improvement obtained using the proposed approach compared to the baseline approach.

In order to investigate the significance in differences of accuracy rate obtained for the proposed model and the baseline approach, the results from Table 9 were used. Two dependent samples were created—one containing the results for the proposed model and one containing the results for the baseline approach. Each sample had a cardinality equal to 13 observations—results obtained for different data sets and number of local tables. The Wilcoxon test confirmed that differences in the accuracy rate between these two groups are significant, with p=0.005.

**Figure 6:**
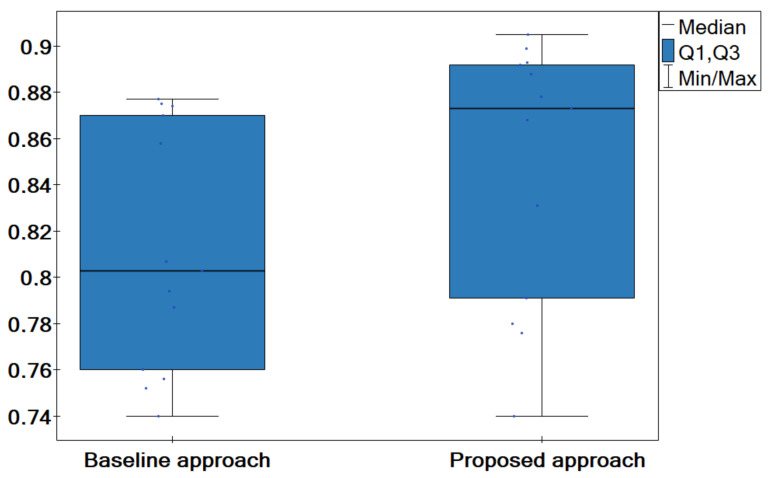
Box-plot chart with (median, the first quartile—Q1, the third quartile—Q3) the value of accuracy rate acc for the proposed model and the baseline approach.

Additionally, a comparative box-plot chart for the accuracy rate values was created (Figure 6). We can observe an increase in accuracy rate when the proposed model is used. Both the box alignment and the median itself are significantly higher when the proposed model is employed.

Furthermore, we also analyzed the time needed to generate decision trees in both approaches. In the baseline method, the time needed to generate trees directly from local tables was investigated, and in the proposed approach the time required to generate trees from aggregated tables was considered. Table 10 shows the execution times of the decision tree generation algorithms in the baseline approach and with coalitions.

The differences in execution times are notably significant. The proposed model has significantly lower time complexity. This is due to the fact that with the proposed approach—coalitions creation—a smaller number of trees is created than when decision trees are generated based on each local table separately. This results in the significantly reduced execution time of making a final decision based on dispersed data.

**Figure 7:**
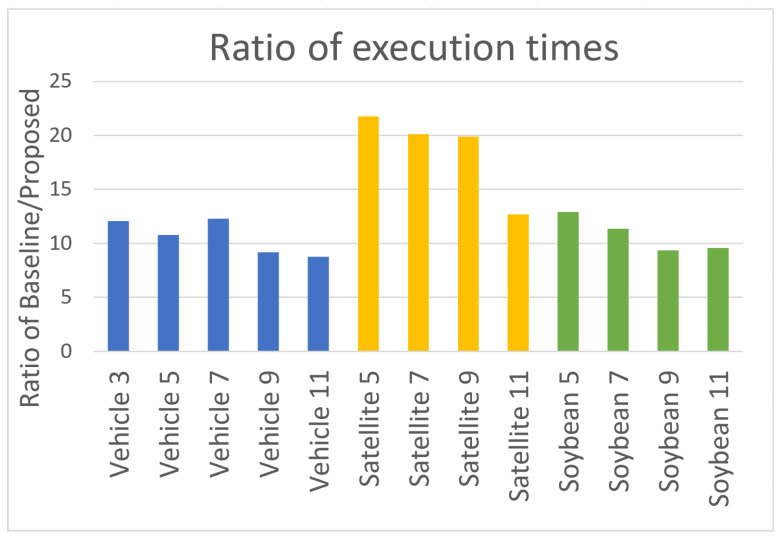
Ratio of execution times of the algorithms implementing the baseline approach and the approach with coalitions.

Figure 7 illustrates the ratio of execution times of the baseline approach to the proposed approach. As can be seen for the Satellite data set, in some cases, the proposed approach exhibits an execution time more than 20-fold faster than the baseline approach. In general, it can be seen that for the largest data set (Satellite) the execution acceleration is the most significant.

In addition, for a smaller degree of dispersion—smaller number of local tables—the reduction in execution time using the proposed approach is greater than for data with a larger degree of dispersion—greater number of local tables. This is due to the fact that for a larger degree of dispersion, there is also a greater number of coalitions generated using the Pawlak analysis model (as can be seen in Table 8).

All experiments were performed on a portable computer with the following technical specifications:

- AMD Ryzen 54,600 h CPU,
- 32 GB RAM Memory,
- Microsoft Windows 11 Operating System.

The code used for the analyzed approaches has been implemented in Python and all data-related calculations have been saved in a text document. Decision trees were built using the function implemented in the Scikit-learn library *tree.DecisionTreeClassifier(criterion = “gini”)*. In all cases, the Gini index was used. The postpruning and prepruning methods were intentionally not applied, since the main goal of this study focused on analyzing how building coalitions of tables using the Pawlak conflict analysis model affects classification quality and model running time. Combining local tables into aggregated tables was shown to significantly improve classification quality. In addition, it also reduces the number of generated trees and thus reduces the time complexity of the method.

## 4. Discussion

The paper proposes a new method for classification based on dispersed data. This method is used when the same set of conditional attributes occurs in all local tables. It should be noted that the conditional attributes can be of different types—both qualitative and quantitative. Sets of objects in local tables can be diversified. Indeed, we do not consider the possibility of examining whether identical objects occur in different local tables. The main idea behind this method is the aggregation of tables that store similar values on conditional attributes. In order to determine which tables should be aggregated, a new method for generating characteristics of values stored in tables and a new method for using the Pawlak conflict analysis model are proposed. Next, a method for defining aggregated tables and a method for final decision-making are defined. It was shown that the proposed method brings a significant improvement in the quality of classification obtained based on dispersed data compared to the approach when aggregation of tables and formation of coalitions are not considered.

The main advantages of the proposed approach are:

- The proposed method guarantees higher quality of classification in comparison with cases where conflict analysis and creation of coalitions are not used.
- The proposed method has less time complexity than methods where coalitions are not considered.
- Combining several similar tables—aggregation of tables into one—increases readability of the model. One decision tree generated based on an aggregated table provides better readability and possibility to interpret the described concepts than several trees generated independently from local tables.

The main limitations of the proposed approach are:

- The proposed model in the current stage of development is dedicated only to a set of local tables with the same sets of conditional attributes.
- Although with the proposed model, the readability of the system is increased by aggregating local tables, we still have not achieved full interpretability of the results. The final classifier consists of a set of decision trees.
- In the proposed approach, it is necessary to exchange data and make them available. The proposed model will not be suitable for dispersed data in which data protection and privacy is a priority.

**Figure 8:**
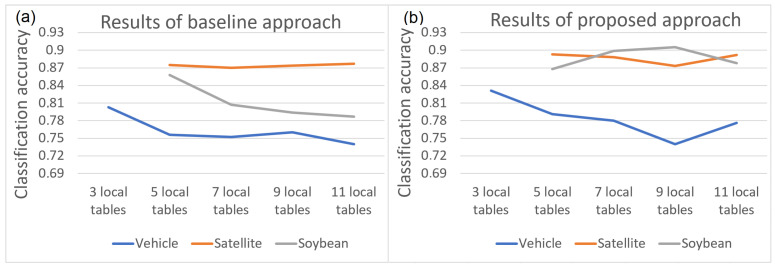
Classification of accuracy values in relation to the number of local tables: (**a**) for the baseline approach (**b**) for the approach with coalitions.

There are practically no parameters in the proposed model, since the Pawlak model has no parameters, and the decision trees were built without prepruning or postpruning (this will be implemented in the next stage of the future work). The only parameter we can consider is the degree of data dispersion. The decision tables were dispersed to varying degrees into 3, 5, 7, 9 and 11 decision tables. The dispersion was performed in relation to the objects in stratified mode and ensuring the number of objects in the local tables remains equal. Figure 8 shows the function of classification accuracy values in relation to the number of local tables.

In the case of the baseline method for both the Soybean and the Vehicle data sets, an increase in the degree of data dispersion results in a deterioration of classification accuracy. For the Landsat Satellite data set, this relation is not observed. For the proposed approach, only for the Vehicle set can it be stated that an increase in the degree of dispersion affects the deterioration of classification accuracy. For the Soybean data set, the proposed method eliminates the negative effect of high dispersion on classification accuracy. Thus, it can be concluded that the use of the proposed approach allows improvement in the quality of classification, especially in the case of high dispersion where many local tables occur. In other words, the proposed model generally improves the quality of classification, but is particularly useful for data dispersed over a large number of local tables.

## 5. Conclusions

A new classification approach based on dispersed data was proposed in this paper. The main innovation lies in the proposal of a method that combines local decision tables into an aggregated table. For this purpose, a method based on the Pawlak conflict analysis model was proposed. The new approach was shown to improve both the quality of classification and the running time.

In future work, we plan to:

- use other classification models different from decision tree to build classifiers based on aggregated tables,
- conduct research on the impact of tree optimization—prepruning and postpruning—on the classification quality of the model,
- extend the proposed model to cases where only parts of the conditional attributes are shared between local tables.

## Figures and Tables

**Table 1:** Comparison of the new approach with the approach proposed in the papers [32,33,34].

	New Proposed Approach	Approach Proposed in the Papers [32,33,34]
**System’s Structure**	**Static**	**Dynamic**
Changeability of coalitions	Coalitions of local tables determined only once regardless of the object that is being classified.	Coalitions of local tables determined for each classified object from scratch.
Basis for coalitions designation	Information system in Pawlak model created based on characteristics of values stored in local tables. So coalitions are created based on conditional attributes’ values occurring in local tables.	Information system in Pawlak model created based on prediction vectors generated for the classified object.
Definition of aggregated table for one coalition	Aggregated table is defined by a sum of objects.	Aggregated table is defined by the approximated method for the aggregation of decision tables—computationally complex.
Base classifiers	Decision tree, CART	k–nearest neighbor classifier
Constraints on local tables	The same conditional attributes in all local tables.	None

**Table 2:** Local tables used in the example.

$U_{1}$	$a_{1}$	$a_{2}$	$a_{3}$	$a_{4}$	$a_{5}$	***d***
$x_{1}$	1	0	2	0	0	$d_{2}$
$x_{2}$	2	1	0	1	0	$d_{2}$
$x_{3}$	0	0	1	2	2	$d_{1}$
$x_{4}$	2	1	1	1	1	$d_{1}$
$x_{5}$	1	2	0	1	2	$d_{2}$
$U_{2}$	$a_{1}$	$a_{2}$	$a_{3}$	$a_{4}$	$a_{5}$	d
$x_{1}$	0	2	1	0	0	$d_{2}$
$x_{2}$	2	1	2	1	2	$d_{1}$
$x_{3}$	2	0	0	2	1	$d_{2}$
$x_{4}$	1	1	2	0	0	$d_{2}$
$x_{5}$	2	0	2	1	1	$d_{1}$
$U_{3}$	$a_{1}$	$a_{2}$	$a_{3}$	$a_{4}$	$a_{5}$	d
$x_{1}$	1	1	0	2	2	$d_{1}$
$x_{2}$	1	1	2	0	1	$d_{1}$
$x_{3}$	2	0	1	2	1	$d_{2}$
$x_{4}$	0	2	0	2	0	$d_{2}$
$x_{5}$	2	0	2	1	2	$d_{2}$
$U_{4}$	$a_{1}$	$a_{2}$	$a_{3}$	$a_{4}$	$a_{5}$	d
$x_{1}$	1	0	0	2	2	$d_{1}$
$x_{2}$	2	1	0	1	0	$d_{2}$
$x_{3}$	0	2	1	2	2	$d_{2}$
$x_{4}$	2	0	2	1	1	$d_{1}$
$x_{5}$	1	2	0	1	1	$d_{2}$

**Table 3:** Averages ${\bar{Val}}_{a_{j}}^{i}$, i∈{1,…,4},j∈{1,…,5}.

Local Table	$a_{1}$	$a_{2}$	$a_{3}$	$a_{4}$	$a_{5}$
$D_{1}$	${\bar{Val}}_{a_{1}}^{1}=1.2$	${\bar{Val}}_{a_{2}}^{1}=0.8$	${\bar{Val}}_{a_{3}}^{1}=0.8$	${\bar{Val}}_{a_{4}}^{1}=1$	${\bar{Val}}_{a_{5}}^{1}=1$
$D_{2}$	${\bar{Val}}_{a_{1}}^{2}=1.4$	${\bar{Val}}_{a_{2}}^{2}=0.8$	${\bar{Val}}_{a_{3}}^{2}=1.4$	${\bar{Val}}_{a_{4}}^{2}=0.8$	${\bar{Val}}_{a_{5}}^{2}=0.8$
$D_{3}$	${\bar{Val}}_{a_{1}}^{3}=1.2$	${\bar{Val}}_{a_{2}}^{3}=0.8$	${\bar{Val}}_{a_{3}}^{3}=1$	${\bar{Val}}_{a_{4}}^{3}=1.4$	${\bar{Val}}_{a_{5}}^{3}=1.2$
$D_{4}$	${\bar{Val}}_{a_{1}}^{4}=1.2$	${\bar{Val}}_{a_{2}}^{4}=1$	${\bar{Val}}_{a_{3}}^{4}=0.6$	${\bar{Val}}_{a_{4}}^{4}=1.4$	${\bar{Val}}_{a_{5}}^{4}=1.2$
Global	${\bar{Val}}_{a_{1}}=1.25$	${\bar{Val}}_{a_{2}}=0.85$	${\bar{Val}}_{a_{3}}=0.95$	${\bar{Val}}_{a_{4}}=1.15$	${\bar{Val}}_{a_{5}}=1.05$
metrics	$SD_{a_{1}}=0.087$	$SD_{a_{2}}=0.087$	$SD_{a_{3}}=0.296$	$SD_{a_{4}}=0.260$	$SD_{a_{5}}=0.166$

**Table 4:** Information system.

*U*	$a_{1}$	$a_{2}$	$a_{3}$	$a_{4}$	$a_{5}$
$D_{1}$	0	0	0	0	0
$D_{2}$	1	0	1	−1	−1
$D_{3}$	0	0	0	0	0
$D_{4}$	0	1	−1	0	0

**Table 5:** Function values.

	$D_{1}$	$D_{2}$	$D_{3}$	$D_{4}$
$D_{1}$				
$D_{2}$	0.8			
$D_{3}$	0	0.8		
$D_{4}$	0.4	1.0	0.4	

**Table 6:** Aggregated local tables.

${U_{1}}^{\mathrm{aggr}}$	$a_{1}$	$a_{2}$	$a_{3}$	$a_{4}$	$a_{5}$	***d***
$x_{1}^{aggr}$	1	0	2	0	0	$d_{2}$
$x_{2}^{aggr}$	2	1	0	1	0	$d_{2}$
$x_{3}^{aggr}$	0	0	1	2	2	$d_{1}$
$x_{4}^{aggr}$	2	1	1	1	1	$d_{1}$
$x_{5}^{aggr}$	1	2	0	1	2	$d_{2}$
$x_{6}^{aggr}$	1	1	0	2	2	$d_{1}$
$x_{7}^{aggr}$	1	1	2	0	1	$d_{1}$
$x_{8}^{aggr}$	2	0	1	2	1	$d_{2}$
$x_{9}^{aggr}$	0	2	0	2	0	$d_{2}$
$x_{10}^{aggr}$	2	0	2	1	2	$d_{2}$
$x_{11}^{aggr}$	1	0	0	2	2	$d_{1}$
$x_{12}^{aggr}$	2	1	0	1	0	$d_{2}$
$x_{13}^{aggr}$	0	2	1	2	2	$d_{2}$
$x_{14}^{aggr}$	2	0	2	1	1	$d_{1}$
$x_{15}^{aggr}$	1	2	0	1	1	$d_{2}$
${U_{2}}^{\mathrm{aggr}}$	$a_{1}$	$a_{2}$	$a_{3}$	$a_{4}$	$a_{5}$	d
$x_{1}^{aggr}$	0	2	1	0	0	$d_{2}$
$x_{2}^{aggr}$	2	1	2	1	2	$d_{1}$
$x_{3}^{aggr}$	2	0	0	2	1	$d_{2}$
$x_{4}^{aggr}$	1	1	2	0	0	$d_{2}$
$x_{5}^{aggr}$	2	0	2	1	1	$d_{1}$

**Table 7:** Data set characteristics.

Data Set	# The Training Set	# The Test Set	# Conditional Attributes	# Decision Classes
Vehicle Silhouettes	592	254	18	4
Landsat Satellite	4435	2000	36	7
Soybean	307	376	35	19

**Table 8:** Coalitions generated using the Pawlak conflict analysis model for dispersed data. LT denotes local table.

Data Set	No. of Local Tables	Coalitions
Vehicle	3	{LT1,LT3},{LT2}
	5	{LT2,LT3,LT4},{LT4,LT5},{LT1}
	7	{LT1,LT3,LT5,LT6,LT7},{LT2},{LT4}
	9	{LT1,LT3,LT4,LT9},{LT3,LT4,LT5,LT6},{LT3,LT4,LT5,LT9},
		{LT2,LT3,LT4,LT9},{LT7,LT8}
	11	{LT2,LT4,LT5,LT8},{LT2,LT5,LT7,LT8},
		{LT2,LT5,LT6,LT8},{LT1,LT9},{LT8,LT9},{LT3,LT10},{LT11}
Satellite	3	NO COALITIONS
	5	{LT1,LT4},{LT2},{LT3},{LT5}
	7	{LT1,LT4,LT6,LT7},{LT3,LT6},{LT2},{LT5}
	9	{LT1,LT4,LT5,LT6,LT9},{LT3,LT4,LT5},{LT2},{LT7},{LT8}
	11	{LT1,LT2,LT7,LT10},{LT1,LT2,LT7,LT11},{LT2,LT6,LT7,LT10},
		{LT2,LT3,LT7,LT9},{LT2,LT4,LT7},
		{LT5,LT9},{LT5,LT11},{LT8}
Soybean	3	NO COALITIONS
	5	{LT2,LT4},{LT1},{LT5},{LT3}
	7	{LT2,LT3,LT5},{LT1,LT3},{LT5,LT7},{LT2,LT4},{LT6}
	9	{LT1,LT2,LT4},{LT1,LT2,LT5},{LT1,LT5,LT6},{LT1,LT3,LT5},
		{LT1,LT9},{LT8,LT9},{LT7}
	11	{LT1,LT4,LT6,LT7,LT8,LT9},{LT1,LT4,LT6,LT7,LT9,LT10},
		{LT1,LT4,LT7,LT8,LT9,LT11},{LT1,LT4,LT7,LT9,LT10,LT11},
		{LT4,LT5,LT6,LT7,LT9,LT10},{LT2},{LT3}

**Table 9:** Results of classification accuracy acc, classification ambiguity accuracy $acc_{ONE}$ and the average number of generated decisions set $\bar{d}$ for all dispersed data sets.

Data Set	No. of Local Tables	Baseline Approach $\mathrm{acc}/{\mathrm{acc}}_{\mathrm{ONE}}/\bar{d}$	Proposed Approach $\mathrm{acc}/{\mathrm{acc}}_{\mathrm{ONE}}/\bar{d}$
Vehicle	3	0.803/0.673/1.268	**0.831**/0.496/1.409
	5	0.756/0.677/1.094	**0.791**/0.709/1.173
	7	0.752/0.681/1.114	**0.780**/0.669/1.228
	9	**0.760**/0.693/1.098	0.740/0.685/1.075
	11	0.740/0.673/1.087	**0.776**/0.728/1.051
Satellite	5	0.875/0.839/1.053	**0.893**/0.820/1.099
	7	0.870/0.841/1.040	**0.888**/0.822/1.093
	9	**0.874**/0.847/1.035	0.873/0.841/1.045
	11	0.877/0.850/1.034	**0.892**/0.857/1.042
Soybean	5	0.858/0.784/1.142	**0.868**/0.791/1.132
	7	0.807/0.716/1.135	**0.899**/0.834/1.074
	9	0.794/0.703/1.152	**0.905**/0.875/1.037
	11	0.787/0.723/1.108	**0.878**/0.855/1.064
Average		0.812/0.746/1.105	**0.847**/0.768/1.117

**Table 10:** Execution times of the decision tree generation algorithms in the base approach and with coalitions.

Data Set	No. of Local Tables	Baseline Approach Time [s]	Proposed Approach Time [s]	Ratio $\frac{\mathrm{Baseline}}{\mathrm{Proposed}}$
Vehicle	3	41.258	3.423	12.05
	5	46.694	4.332	10.78
	7	52.810	4.294	12.30
	9	61.634	6.704	9.19
	11	68.064	7.760	8.77
Satellite	5	3044.087	139.973	21.75
	7	3228.569	160.59	20.10
	9	3497.267	175.614	19.91
	11	3658.961	288.654	12.68
Soybean	5	58.542	4.538	12.90
	7	63.733	5.610	11.36
	9	72.051	7.714	9.34
	11	82.072	8.560	9.59

## Data Availability

Publicly available data sets were analyzed in this study. These data can be found here: [38]. One data set has been artificially generated and a description of the process behind the artifical generation is presented in the paper.

## References

[1] Czarnowski I., Jȩdrzejowicz P. (2015). Ensemble online classifier based on the one-class base classifiers for mining data streams. Cybern. Syst..

[2] Verbraeken J., Wolting M., Katzy J., Kloppenburg J., Verbelen T., Rellermeyer J.S. (2020). A survey on distributed machine learning. ACM Comput. Surv..

[3] Guo Y., Zhao R., Lai S., Fan L., Lei X., Karagiannidis G.K. (2022). Distributed machine learning for multiuser mobile edge computing systems. IEEE J. Sel. Top. Signal Process..

[4] Ma C., Li J., Shi L., Ding M., Wang T., Han Z., Poor H.V. (2022). When federated learning meets blockchain: A new distributed learning paradigm. IEEE Comput. Intell. Mag..

[5] Xiao M., Skoglund M. (2022). Coding for Large-Scale Distributed Machine Learning. Entropy.

[6] Rodríguez-Barroso N., Stipcich G., Jiménez-López D., Ruiz-Millán J.A., Martínez-Cámara E., González-Seco G., Luzóna M.V., Veganzones M.A., Herrera F. (2020). Federated learning and differential privacy: Software tools analysis, the sherpa. ai fl framework and methodological guidelines for preserving data privacy. Inf. Fusion.

[7] Yang Q., Liu Y., Chen T., Tong Y. (2019). Federated machine learning: Concept and applications. ACM Trans. Intell. Syst. Technol. (TIST).

[8] Ng W.W., Zhang J., Lai C.S., Pedrycz W., Lai L.L., Wang X. (2018). Cost-sensitive weighting and imbalance-reversed bagging for streaming imbalanced and concept drifting in electricity pricing classification. IEEE Trans. Ind. Inform..

[9] Czarnowski I. (2022). Weighted Ensemble with one-class Classification and Over-sampling and Instance selection (WECOI): An approach for learning from imbalanced data streams. J. Comput. Sci..

[10] Pławiak P., Abdar M., Pławiak J., Makarenkov V., Acharya U.R. (2020). DGHNL: A new deep genetic hierarchical network of learners for prediction of credit scoring. Inf. Sci..

[11] Gupta O., Raskar R. (2018). Distributed learning of deep neural network over multiple agents. J. Netw. Comput. Appl..

[12] Alsahaf A., Petkov N., Shenoy V., Azzopardi G. (2022). A framework for feature selection through boosting. Expert Syst. Appl..

[13] Hashemi A., Dowlatshahi M.B., Nezamabadi-Pour H. (2022). Ensemble of feature selection algorithms: A multi-criteria decision-making approach. Int. J. Mach. Learn. Cybern..

[14] Ślȩzak D., Janusz A. (2011). Ensembles of bireducts: Towards robust classification and simple representation. Proceedings of the International Conference on Future Generation of Information Technology (FGIT).

[15] Kozak J. (2019). Decision Tree and Ensemble Learning Based on Ant Colony Optimization.

[16] Tüysüzoğlu G.Ö.K.S.U., Birant D. (2020). Enhanced bagging (eBagging): A novel approach for ensemble learning. Int. Arab. J. Inf. Technol..

[17] Batra S., Khurana R., Khan M.Z., Boulila W., Koubaa A., Srivastava P. (2022). A Pragmatic Ensemble Strategy for Missing Values Imputation in Health Records. Entropy.

[18] Nam G., Yoon J., Lee Y., Lee J. (2021). Diversity matters when learning from ensembles. Adv. Neural Inf. Process. Syst..

[19] Ortega L.A., Cabañas R., Masegosa A. Diversity and generalization in neural network ensembles. Proceedings of the International Conference on Artificial Intelligence and Statistics.

[20] Kashinath S.A., Mostafa S.A., Mustapha A., Mahdin H., Lim D., Mahmoud M.A., Mohammed M.A., Al-Rimy B.A.S., Fudzee M.F., Yang T.J. (2021). Review of data fusion methods for real-time and multi-sensor traffic flow analysis. IEEE Access.

[21] Kuncheva L.I. (2014). Combining Pattern Classifiers: Methods and Algorithms.

[22] Liu L., Zhang J., Song S.H., Letaief K.B. Client-edge-cloud hierarchical federated learning. Proceedings of the ICC 2020-2020 IEEE International Conference on Communications (ICC).

[23] Zhou C., Zhang H., Valdebenito M.A., Zhao H. (2022). A general hierarchical ensemble-learning framework for structural reliability analysis. Reliab. Eng. Syst. Saf..

[24] Gholizadeh N., Musilek P. (2021). Distributed Learning Applications in Power Systems: A Review of Methods, Gaps, and Challenges. Energies.

[25] Tang M., Liao H., Mi X., Lev B., Pedrycz W. (2021). A hierarchical consensus reaching process for group decision making with noncooperative behaviors. Eur. J. Oper. Res..

[26] Dai T., Sycara K., Zheng R. (2021). Agent reasoning in AI-powered negotiation. Handbook of Group Decision and Negotiation.

[27] Wyai L.C., WaiShiang C., Lu M.V.A. (2018). Agent negotiation patterns for multi agent negotiation system. Adv. Sci. Lett..

[28] Pawlak Z. (2005). Some remarks on conflict analysis. Eur. J. Oper. Res..

[29] Pawlak Z. Conflict analysis. Proceedings of the Fifth European Congress on Intelligent Techniques and Soft Computing (EUFIT’97).

[30] Tong S., Sun B., Chu X., Zhang X., Wang T., Jiang C. (2021). Trust recommendation mechanism-based consensus model for Pawlak conflict analysis decision making. Int. J. Approx. Reason..

[31] Yao Y. (2019). Three-way conflict analysis: Reformulations and extensions of the Pawlak model. Knowl. Based Syst..

[32] Przybyła-Kasperek M. (2022). Study of selected methods for balancing independent data sets in k-nearest neighbors classifiers with Pawlak conflict analysis. Appl. Soft Comput..

[33] Przybyła-Kasperek M. (2020). Coalitions’ Weights in a Dispersed System with Pawlak Conflict Model. Group Decis. Negot..

[34] Przybyła-Kasperek M. (2019). Three conflict methods in multiple classifiers that use dispersed knowledge. Int. J. Inf. Technol. Decis. Mak..

[35] Breiman L., Friedman J.H., Olshen R.A., Stone C.J. (2017). Classification and Regression Trees.

[36] Przybyła-Kasperek M., Wakulicz-Deja A. (2014). Global decision-making system with dynamically generated clusters. Inform. Sci..

[37] Lamrini B. (2020). Contribution to Decision Tree Induction with Python: A Review. Data Mining—Methods, Applications and Systems.

[38] Asuncion A., Newman D.J. (2007). UCI Machine Learning Repository.

